# Reactivation of the Unconditioned Stimulus Inhibits the Return of Fear Independent of Cortisol

**DOI:** 10.3389/fnbeh.2019.00254

**Published:** 2019-11-12

**Authors:** Shira Meir Drexler, Christian J. Merz, Silke Lissek, Martin Tegenthoff, Oliver T. Wolf

**Affiliations:** ^1^Department of Cognitive Psychology, Institute of Cognitive Neuroscience, Ruhr University Bochum, Bochum, Germany; ^2^Department of Neurology, BG University Hospital Bergmannsheil, Ruhr University Bochum, Bochum, Germany

**Keywords:** extinction, fear conditioning, glucocorticoids, reconsolidation, reinstatement, retrieval

## Abstract

Reconsolidation is the post-retrieval stabilization of memories, a time-limited process during which reactivated (i.e., retrieved) memories can be updated with new information, become stronger or weaker, depending on the specific treatment. We have previously shown that the stress hormone cortisol has an enhancing effect on the reconsolidation of fear memories in men. This effect was specific, i.e., limited to the conditioned stimulus (CS) that was reactivated, and did not generalize to other previously reinforced, but not reactivated CS. Based on these results, we suggested that cortisol plays a critical role in the continuous strengthening of reactivated emotional memories, contributing to their persistence and robustness. In the current study, we aimed to achieve a more generalized reconsolidation enhancement using an alternative reactivation method, i.e., by a low-intensity unconditioned stimulus (UCS) presentation instead of the more common unreinforced CS presentation. In previous studies, UCS reactivation was shown to lead to a more generalized reconsolidation effect. Therefore, we hypothesized that the combination of cortisol treatment and UCS reactivation would lead to an enhanced fear memory reconsolidation, which would generalize from previously reinforced CS to stimuli that resemble it. We tested 75 men in a 3-day fear conditioning paradigm: fear acquisition training on day 1; UCS reactivation/no reactivation and pharmacological treatment (20 mg hydrocortisone/placebo) on day 2; extinction training, reinstatement and test (of original and modified stimuli) on day 3. In contrast to our hypothesis, UCS reactivation prevented the return of fear [observed in skin conductance responses (SCR)] regardless of the pharmacological manipulation: while reinstatement to the original CS was found in the no-reactivation group, both reactivation groups (cortisol and placebo) showed no reinstatement. As the only methodological difference between our previous study and the current one was the reactivation method, we focus on UCS reactivation as the main explanation for these unexpected findings. We suggest that the robust prediction error generated by the UCS reactivation method (as opposed to CS reactivation), combined with the lower UCS intensity, has by itself weakened the emotional value of the UCS, thus preventing the return of fear to the CS that was associated with it. We call for future research to support these findings and to examine the potential of this reactivation method, or variations thereof, as a tool for therapeutic use.

## Introduction

In the late 1960s, Misanin et al. (1968) discovered that already-consolidated memories could once again become susceptible to interruption after their retrieval. This finding stood in contrast to the dominant view on memory at that time, according to which memory consolidation is a one-time process. Decades later, a renewed interest in memory reconsolidation emerged following the work of Nader et al. (2000), which demonstrated memory impairment as a result of post-retrieval administration of protein-synthesis inhibitors. It is estimated that the reactivated (i.e., retrieved) memory can be modified during a temporal window of up to several hours post-retrieval (Schiller et al., 2010; Cahill et al., 2019): it can either be updated with new information (Exton-McGuinness et al., 2015; Haubrich et al., 2015; Hadamitzky et al., 2016), lose (Nader et al., 2000) or gain (Frenkel et al., 2005) its strength. The direction of the effect depends on the exact manipulation (be it pharmacological, behavioral or otherwise) and additional factors (such as memory type; for a review, see Meir Drexler and Wolf, 2017c). Since maladaptive learning and memory processes underlie various psychological disorders (e.g., posttraumatic stress disorder, specific phobias, and addictions; Coles and Heimberg, 2002; Hyman et al., 2006), the increasing knowledge on memory reconsolidation can significantly contribute to the understating and treatment of these disorders (Soeter and Kindt, 2015; Kindt and van Emmerik, 2016; Elsey and Kindt, 2017; Meir Drexler and Wolf, 2018). The current study, therefore, aimed to reveal the role of cortisol in the strengthening and generalization of reactivated emotional memories.

Cortisol, the end-product of the hypothalamus-pituitary-adrenal (HPA) axis, represents a major candidate for the modification of memory reconsolidation. It is a glucocorticoid (GC), which is secreted in a circadian rhythm (i.e., higher levels upon awakening; Pruessner et al., 1997) and following exposure to a stressful event (Joëls et al., 2006; Joëls and Baram, 2009). Cortisol administration or stress exposure have timing-dependent effects on learning and memory processes. Typically, both stress and cortisol enhance memory consolidation and impair retrieval (Wolf et al., 2016; Meir Drexler and Wolf, 2017a; de Quervain et al., 2017). Their effects on memory reconsolidation, however, may be robust but are sometimes mixed with regard to their direction. Animal studies have reported mainly an impairing effect of stress or corticosterone (the rodent equivalent of cortisol) treatment on memory reconsolidation (Wang et al., 2008; Yang et al., 2013). However, GC antagonists were reported as having impairing effects on reconsolidation as well (Pitman et al., 2011). Conflicting findings were also found in human studies (see Zhao et al., 2009; Schwabe and Wolf, 2010; Coccoz et al., 2011; Meir Drexler et al., 2015; Meir Drexler and Wolf, 2017b). In general, the effects of GCs on learning and memory processes depend on various factors other than the targeted memory phase alone (Sandi and Pinelo-Nava, 2007; Akirav and Maroun, 2013; Meir Drexler and Wolf, 2017c; Meir Drexler et al., 2019), and so conflicting findings in reconsolidation studies are no exception. These mixed results are likely the outcome of the large methodological variations between studies (Lonsdorf et al., 2017).

We have previously investigated the effects of cortisol on the reconsolidation of fear memories in men and women. In our paradigm, participants learned to associate two conditioned stimuli (CS1+, CS2+) with an electrical shock and a conditioned stimulus (CS) that was not paired with it (CS−). One day later, they received either cortisol or placebo and retrieved the memory of one of the stimuli by an unreinforced presentation of CS1+. We found that cortisol enhanced the reconsolidation of fear memories selectively to the CS1+ and only in men (Meir Drexler et al., 2015), while having no effect in women (possibly due to interaction with sex hormones during the menstrual cycle and following oral contraceptive use, see Meir Drexler et al., 2016). We thus suggested that cortisol plays a critical role in the continuous strengthening of emotional memories, contributing to their persistence, at least in men. Notably, the cortisol-dependent reconsolidation enhancement we found was limited to the memory that was reactivated and did not generalize to the CS2+ that was not reactivated. A specific reconsolidation effect was also observed in other studies that used similar reactivation methods, i.e., by an unreinforced CS presentation (Schiller et al., 2010; Hupbach and Dorskind, 2014) albeit not in all (Soeter and Kindt, 2015). Since real-life aversive memories are not associated with a single cue, a generalized reconsolidation effect is more therapeutically desirable in its ability to prevent relapses (Soeter and Kindt, 2015). A non-specific effect might also be more ecologically valid in revealing the underlying mechanism of emotional memories, which are often generalized over time (Vervliet et al., 2013; Dunsmoor and Murphy, 2015; Pollack et al., 2018; Meir Drexler et al., 2019).

In this study, we aimed to extend our previous findings on the enhancing effects of cortisol on the reconsolidation of fear memories in men (Meir Drexler et al., 2015) by creating a more generalized reconsolidation effect. For this aim, we used an alternative reactivation method that involves a low-intensity UCS presentation instead of the more commonly-used unreinforced CS reactivation. Previous studies demonstrate that the UCS reactivation procedure leads to a generalized reconsolidation effect (Liu et al., 2014; Luo et al., 2015; Thompson and Lipp, 2017). For instance, Liu et al. (2014) showed that a single weaker presentation of the UCS before extinction reduced the return of fear in both humans and rodents. Unlike CS reactivation, the effects of UCS reactivation also generalized to stimuli that were previously conditioned but were not reactivated before extinction training. In rodents, similar generalized effects were found in a contextual paradigm for drug-seeking behavior with cocaine priming as a trigger for UCS reactivation (Luo et al., 2015). These findings suggest that, while the CS reactivation paradigm selectively destabilizes only the reactivated CS+, weaker UCS reactivation destabilizes all CS+ that were previously related to it regardless of their direct reactivation (Liu et al., 2014, 2019; Luo et al., 2015; Thompson and Lipp, 2017). It is currently unclear whether the generalized reconsolidation effect triggered by UCS can also influence stimuli that were never directly conditioned but share similarity to the CS+.

Based on these findings, we hypothesized that the combination of cortisol and UCS reactivation would lead to an enhanced and generalized fear memory reconsolidation (compared to reactivation with placebo or cortisol administration without reactivation). This effect should be reflected in higher conditioned responses after reinstatement for both the original CS+ and stimuli that resemble it.

## Materials and Methods

### Participants

We previously found differences between men and women in the effects of cortisol on fear memory reconsolidation (Meir Drexler et al., 2015, 2016). These differences may result from the influence of sex hormones on emotional learning and memory (e.g., during the female menstrual cycle or following hormonal contraceptives intake; Merz et al., 2018; Raeder et al., 2019; Velasco et al., 2019). For this reason, we limited our sample to men only.

Seventy-five men participated in this study. Since this experiment was not intended as a direct replication of our previous CS reactivation study (Meir Drexler et al., 2015) but as an investigation of the alternative UCS reactivation method, we did not include any CS reactivation groups. In line with previous publications (Kindt et al., 2009; Meir Drexler et al., 2015, 2016), the participants were thus randomly assigned to one of three groups: UCS reactivation + cortisol (RE+CORT), UCS reactivation + placebo (RE), and no reactivation + cortisol (CORT). In our previous study in men (Meir Drexler et al., 2015), the interaction CS × Group revealed a significantly higher reinstatement for the reactivated stimulus CS1+ in the target group RE+CORT. The interaction effect was found to be medium (CS × Group interaction corresponded to an effect size *f* of 0.39). We calculated the power to find a similar interaction effect in the current study using G*Power for Windows 3.1.9.2. (Faul et al., 2009). The power of our sample to detect a medium interaction effect was larger than 97%.

The participants were aged 18–39 years (*M* = 25.43; *SD* = 4.54) and had a body mass index (BMI) of 18–29 kg/m^2^ (*M* = 23.71; *SD* = 2.22). They reported to be non-smokers, healthy (i.e., no somatic, endocrine, psychiatric or neurological diseases) and with no regular medication intake. All participants were students to either a bachelor’s or master’s degree at the Ruhr University Bochum, Germany. They were recruited *via* advertisements on the campus and received a financial reimbursement of 50€ (approximately 57$) for participation. The study was approved by the ethics committee of the Faculty of Medicine at the Ruhr University Bochum (registration number: 16-5788, approval date: 11/8/2016) and was conducted in accordance with the Declaration of Helsinki. At the beginning of the first testing session, all participants signed the informed consent in the presence of the research experimenter.

### Stimuli

#### Conditioned Stimuli (CS)

**Figure 1:**
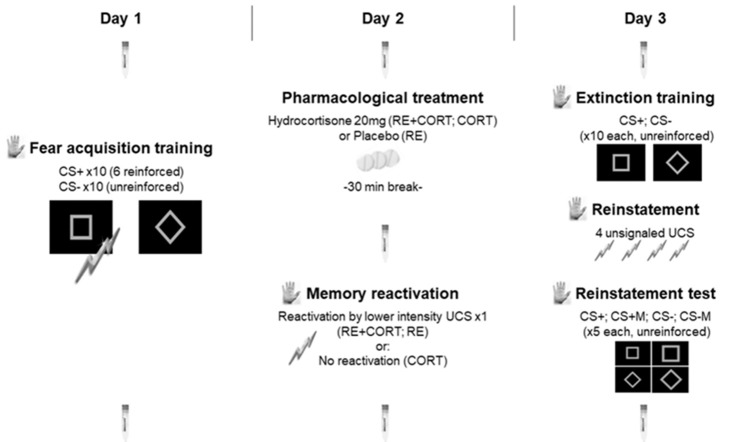
Experimental timeline. The testing was conducted on three consecutive days separated by 24-h intervals: fear acquisition training on day 1; pharmacological treatment and memory reactivation on day 2; extinction training, reinstatement and reinstatement test on day 3. The procedure was identical for the three groups on days 1 and 3 and differed between the three groups only on day 2, in which memory was either reactivated (RE+CORT, RE groups) or not reactivated (CORT group) following the administration of cortisol (RE+CORT, CORT) or placebo (RE). Skin conductance responses (SCR; illustrated by the palm) served as a measure of conditioned fear, and were recorded during all experimental phases. Seven saliva samples (illustrated by the saliva collecting devices) were used to assess salivary cortisol, and were collected during the three experimental days. CS, conditioned stimulus; CSM, conditioned stimulus modified. Bolts, representation of the unconditioned stimulus (UCS).

Two geometrical shapes (a square and a rhombus) in gray color and identical luminescence were counterbalanced between participants as CS+ and CS− (Meir Drexler et al., 2015, 2016; Meir Drexler and Wolf, 2017b; Haaker et al., 2019). For the reinstatement test on day 3, modified versions of the respective CS+ and CS− were also presented (either larger with thicker borders or smaller with thinner borders; see Figure 1). Stimuli were presented for 8 s against a black background in an 800 × 600 pixel resolution on a 19-inch computer screen, at a distance of approximately 50 cm of the participant’s head.

#### Unconditioned Stimulus (UCS)

An electric shock was used as UCS (Meir Drexler et al., 2015, 2016; Meir Drexler and Wolf, 2017b; Haaker et al., 2019). A constant voltage stimulator (STM200; BIOPAC Systems Inc., Goleta, CA, USA) was used to deliver transcutaneous electrical stimulation (100 ms) through two Ag/AgCl electrodes (0.5 cm^2^ surface) filled with isotonic (0.05 M NaCl) electrolyte medium (Synapse Conductive Electrode Cream, Kustomer Kinetics Inc., Arcadia, CA, USA) placed on the left shin. The participants were asked to rate the UCS on a 1–7 scale (1 = not uncomfortable; 7 = very uncomfortable) at four time points during the study: after the shock workup procedure and after acquisition training on day 1, after reactivation (only reactivation groups) on day 2, and after reinstatement test on day 3.

### Instructions

Before each of the experimental phases [see “Conditioning Procedure (Days 1–3)” section], the participants were instructed (both orally and in a written form) that they may or may not receive an electrical stimulation after the presentation of a visual stimulus. They were encouraged to pay attention to the task and look for any regularities. Such regularities, they were informed, would remain stable during the experiment: if a stimulus was safe, it would always be safe, if a stimulus was paired with the electrical stimulation, this might happen again. Although contingency awareness was not used as exclusion criterion in the study, the participants were asked to report any contingencies they witnessed following each of the phases. This procedure was used to facilitate contingency learning and to prevent participants from expecting contingency reversal. At no point were the participants explicitly informed about the actual CS-UCS contingencies or any change in reinforcement rate.

### Conditioning Procedure (Days 1–3)

For the current study, we adapted the 3-day fear conditioning paradigm from our previous reconsolidation studies (Meir Drexler et al., 2015, 2016; Meir Drexler and Wolf, 2017b). In contrast to our previous publications, in which the memory was reactivated using the unreinforced CS+, here we used a low-intensity UCS as a reminder cue (see Figure 1; Liu et al., 2014).

The test sessions took place in the afternoons (starting between 12:30 pm and 5:45 pm) of three consecutive days: fear acquisition training on day 1; memory reactivation and pharmacological treatment on day 2; extinction training, reinstatement, and reinstatement test on day 3. The individual testing schedules were timed so that there were 24 h (±2 h) between each session to allow memory consolidation after each phase (Dudai, 2012). The participants were asked to avoid alcohol consumption during the three testing days and to refrain from eating, drinking (anything but water) and physical activity 90 min before each testing session. Context can serve as a robust retrieval cue (Bouton, 2002; Kredlow et al., 2016; Meir Drexler et al., 2019), also in interaction with stress (Sazma et al., 2019). In order to reduce the possible effects of context as much as possible, all phases of the 3-day testing schedule (including the learning phases and pill administration) were conducted in the presence of the same experimenter in the same room at approximately the same time of day.

#### Day 1: Fear Acquisition Training

Upon arrival on the first testing day, the participants provided written informed consent. Then, they completed questionnaires regarding demographic data and trait anxiety (State-Trait Anxiety Inventory, STAI-T; Spielberger et al., 1970). After receiving instructions on the task (see above), the participants were attached with electrodes for measuring skin conductance responses (SCR) and shock administration. Following a workup procedure, which was performed to attain a subjective “unpleasant but not painful” shock level, the participants underwent the acquisition procedure. Two conditioned stimuli (CS1+, CS−) were used for differential fear conditioning. The CS+ co-terminated with the UCS in reinforced trials (reinforcement rate: 60%) while the CS− was never reinforced. Each CS was presented ten times in a pseudo-randomized order with the following restrictions: the first and last two stimuli presentations included both a reinforced CS+ and a CS−; each stimulus was presented no more than twice in a row; the reinforced presentations of the CS+ were equally distributed between the first and second half of acquisition. The four randomizations for trial order were counterbalanced between participants. The inter-trial interval (ITI) was 6–8 s.

#### Day 2: Pharmacological Treatment and Reactivation

On the following day, the participants received either cortisol (RE+CORT, CORT groups) or placebo (RE group). To allow a peak in cortisol concentrations, participants from the reactivation groups (RE+CORT, RE groups) were instructed to wait for 30 min. They were then attached to both SCR and shock electrodes and received task instructions (see “Stimuli” section). In order to reactivate the fear memory, a single lower intensity UCS was administered (approximately 80% of the UCS intensity used on day 1; during the UCS presentation, the screen presented a black background). This single presentation of the UCS concluded the learning procedure of this experimental day. The participants from the no-reactivation group (CORT) had no further intervention on day 2 apart from pill intake; they were not attached to either SCR or shock electrodes or interacted with the experimental computer in any task. Participants from the CORT remained in the experimental room for the same amount of time (approximately 45 min) as did the participants from the reactivation groups and were free to engage in any activity (e.g., reading) during this period until the end of the testing day.

####  Day 3: Extinction Training, Reinstatement, and Reinstatement Test

After receiving instructions on the task (see “Instructions” section), the participants were attached with the SCR and shock electrodes. For extinction training, both CS+ and CS− were presented, unreinforced, 10 times each (ITI: 6–8 s). The four pseudo-randomizations for trial order, counterbalanced between participants, were restricted as follows: each stimulus was presented no more than twice in a row; the first and last two stimuli presentations included both CS+ and CS−. Extinction was followed by a 10 s break (screen background: black with a fixation cross). Then, reinstatement comprised the presentation of four unsignaled UCS (screen background: gray; 2, 7, 12, and 17 s interval after each shock). The reinstatement test then followed, consisting of five presentations each of the following stimuli: CS+ (original CS+), CS+M (modified CS+), CS− (original CS−), CS−M (modified CS−). All stimuli were unreinforced; ITI was between 6–8 s. Four pseudo-randomizations for trial order were counterbalanced between participants. Each of the four stimuli was presented in the beginning or at the end of one of the trial randomizations; each of the four stimuli was presented once in every four-trial-block of the reinstatement test.

### Pharmacological Intervention (Day 2)

On day 2, the participants were given an oral dose of 20 mg cortisol (two pills of hydrocortisone 10 mg, Jenapharm) or visually identical placebos (two pills of P Tabletten, Weiss 7 mm, Winthrop). At the end of day 2 (approximately 45 min after pill intake), participants were asked to provide a treatment guess (“cortisol”/“placebo”/“I do not know”).

### Measurements and Assessments

#### Skin Conductance Responses (SCR)

In line with previous human fear conditioning studies (Lonsdorf et al., 2017) and our previous reconsolidation publications (Meir Drexler et al., 2015, 2016; Meir Drexler and Wolf, 2017b), SCR served as a measure of the conditioned fear response. SCR were sampled (sampling rate: 1,000 Hz) using a commercial SCR coupler and amplifying system (MP150 + GSR100C, BIOPAC Systems, Inc.; software: AcqKnowledge 4.2) using Ag/AgCl electrodes (0.5 cm^2^ surface) filled with isotonic (0.05M NaCl) electrolyte medium (Synapse Conductive Electrode Cream, Kustomer Kinetics Inc., Arcadia, CA, USA) placed on the hypothenar of the non-dominant hand. Data were manually scored using a custom-made MATLAB script. The maximal base-to-peak difference in SCR was used as a measure for conditioned (1–8.49 s after CS onset) and unconditioned (8.5–14 s after CS onset) responses. Data was transformed with the natural logarithm to attain a normal distribution.

#### Saliva Sampling

Free salivary cortisol concentrations were used to validate the effects of the pharmacological intervention. Saliva samples were collected using Salivette (Sarstedt, Nuembrecht, Germany) collection devices at seven time-points during the three experimental days. On day 1 and day 3, samples were taken at the beginning and end of the session. On day 2, samples were taken at the beginning of the session (immediately before pill intake), 30 min after the pharmacological treatment (before memory reactivation) and at the end of the session (approximately 45 min after the pharmacological treatment; see Figure 1). The samples were kept at −18°C until biochemical analysis. Salivary cortisol was analyzed on a Synergy2 plate reader (Biotek, USA) using commercial enzyme-linked immunosorbent assays (ELISAs; free cortisol in saliva; Demeditec, Germany) according to the manufacturer’s instructions. Intra- and inter-assay variability were less than 10%.

### Statistical Analyses

All statistical analyses were performed using IBM SPSS Statistics for Windows 20. The statistical significance level was set to *α* = 0.05. Greenhouse-Geisser corrected *P*-values were used if assumptions of sphericity were violated. Significant ANOVAs were followed by Bonferroni-adjusted *post hoc* tests.

## Results

The results are based on a total of 75 participants who completed the three testing days: RE+CORT (*N* = 25), RE (*N* = 25), CORT (*N* = 25). One participant from the CORT group was excluded from the cortisol analysis due to missing data. The following participants were excluded from the SCR analyses due to missing data or technical failure: in extinction analysis, one participant from the RE+CORT group; in reinstatement test analysis, one participant from the RE+CORT group and one participant from the CORT group. No additional exclusion criteria were employed.

The three groups did not significantly differ in STAI-T score (*p* > 0.1; not shown).

### Cortisol Concentrations, UCS Ratings, and Treatment Guess

To confirm a rise in free salivary cortisol concentrations on day 2 after hydrocortisone intake compared to placebo, we ran a repeated-measures ANOVA with the within-subjects factor Time (baseline, 30 min, and 45 min after pill intake) and the between-subjects factor Group (RE+CORT, RE, and CORT). The analysis revealed a significant Time × Group interaction (*F*_(4,142)_ = 5.70, *p* < 0.001). We then re-analyzed each group separately and found a main effect of Time in each of the three groups: RE+CORT (*F*_(2,48)_ = 10.71, *p* < 0.001), RE (*F*_(2,48)_ = 8.86, *p* = 0.001), and CORT (*F*_(2,46)_ = 9.98, *p* < 0.001). Bonferroni corrected *post hoc* analysis revealed that cortisol concentrations were significantly (*p* < 0.005) higher at 30 and 45 min after treatment compared with baseline in the cortisol groups RE+CORT and CORT. The placebo group, in contrast, showed significantly lower cortisol concentrations at 30 and 45 min compared to baseline (*p* < 0.05; Table 1). No significant Time × Group interactions were found on day 1 or day 3 (all *p* > 0.1; not shown). In addition, a MANOVA comparing the cortisol concentrations between the groups (dependent variables: samples 1–7) confirmed that the groups significantly differed only on the second testing day, in the samples that were taken 30 min (*F*_(2,74)_ = 10.95, *p* < 0.001) and 45 min (*F*_(2,74)_ = 9.31, *p* < 0.001) after pill intake. Bonferroni corrected *post hoc* analysis revealed significantly (*p* < 0.001) higher cortisol concentrations in each of the cortisol groups (RE+CORT and CORT) in comparison to the placebo group (RE) in both time points after pill intake (for all other comparisons, *p* > 0.1; not shown).

**Table 1:** Mean (SEM) cortisol concentrations (in nmol/l) on day 2 in the three groups (RE+CORT, RE, CORT) before pill intake, 30 and 45 min after pill intake.

Cortisol concentrations (nmol/l)		Before pill intake	After 30 min	After 45 min
Group	RE + CORT (*n* = 25)	12.84 ± 7.29	189.51 ± 155.27***	255.89 ± 280.04***
	RE (*n* = 25)	13.49 ± 8.38	10.97 ± 8.06*	10.28 ± 7.05**
	CORT (*n* = 24)	16.09 ± 15.53	275.25 ± 319.33**	270.59 ± 306.69**

Note. In the cortisol groups RE+CORT and CORT, cortisol concentrations were higher at 30 and 45 min after treatment compared with baseline. The placebo group (RE), in contrast, showed significantly lower cortisol concentrations at 30 and 45 min compared to baseline. ***p ≤ 0.001; **p ≤ 0.01; *p ≤ 0.05 (*post hoc* pairwise comparisons using the *Bonferroni* correction).

The UCS ratings confirmed that participants in the reactivation groups perceived the lower intensity UCS (80%) during reactivation as less aversive compared with acquisition UCS. A repeated-measures ANOVA with the within-subjects factor time (acquisition rating, reactivation rating) and the between-subjects factor Group (RE+CORT, RE) revealed a significant Time effect (*F*_(1,48)_ = 25.44, *p* < 0.001) and no Group effects or interaction (all *p* > 0.05).

The participants’ treatment guess was analyzed using Fisher’s exact test with the directed answers “placebo” and “cortisol” only (excluding “I do not know”). The results of the treatment guess showed that the participants were unable to identify the substance they had been administered with (Fisher’s exact test: *p* = 0.28). In the placebo group, 14 participants correctly supposed the intake of placebo, but four were mistaken in assuming cortisol. Eight men in the cortisol group correctly indicated to have received cortisol, while 19 mistakenly guessed the intake of placebo. The remaining 30 participants provided no treatment guess at all.

### SCR During the Learning Phases

No group differences were found in the unconditioned response to the reactivation shock on day 2 (comparisons between RE+ and RE+CORT groups) or to the four reinstatement shocks on day 3 (comparisons between the three experimental groups): for all comparisons, *p* > 0.1 (not shown). The following presents the analyses of the conditioned responses.

#### Fear Acquisition Training

**Figure 2:**
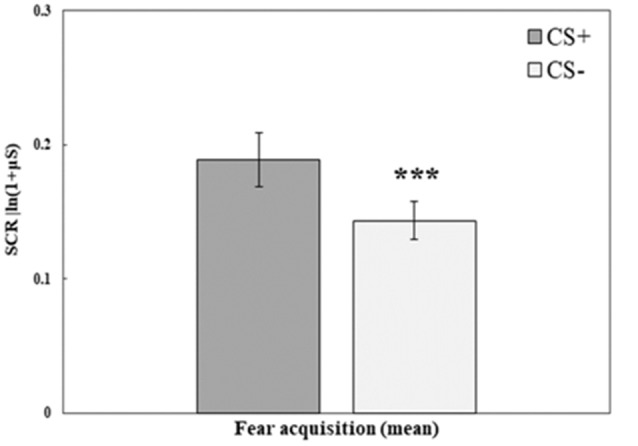
Day 1: Fear acquisition training. Mean (± SEM) SCR to the three conditioned stimuli (mean of 10 trials for CS+ and CS−). SCR to the reinforced CS+ are significantly higher (RE+CORT: 0.16 ± 0.03; RE: 0.17 ± 0.03; CORT: 0.24 ± 0.04) than the SCR to the unreinforced CS− (RE+CORT: 0.10 ± 0.02; RE: 0.14 ± 0.02; CORT: 0.18 ± 0.03; ***p < 0.001), demonstrating successful fear learning. The figure presents all three groups combined (*N* = 75; RE+CORT group: *n* = 25, RE group: *n* = 25, CORT group: *n* = 25) as no interaction and no main effect of group were found.

Fear acquisition training was successful in creating higher SCR to the stimulus that was paired with a shock (CS+) compared with the stimulus that was not paired with a shock (CS−). A repeated-measures ANOVA with the within-subjects factor CS (mean of trials 1–10 for CS+, CS−) and the between-subjects factor Group (RE+CORT, RE, CORT) revealed a significant CS effect (*F*_(1,72)_ = 17.23, *p* < 0.001; for all additional comparisons, including Group, *p* > 0.1). Figure 2 illustrates the differences between the two stimuli during fear acquisition training. As no interaction and no main effect of group were found, the three groups are combined.

#### Extinction Training

**Figure 3:**
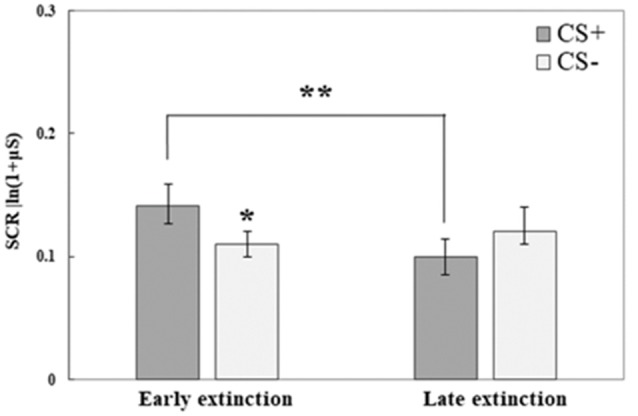
Day 3: Fear extinction training took place on the third testing day. Here, both conditioned stimuli (CS+, CS−) were presented (both not reinforced) for 10 times each. This graph illustrates mean (± SEM) SCR to both stimuli at early extinction (trials 1–5) vs. late extinction (trials 6–10). The significant difference between CS+ and CS− during early extinction (*p < 0.05) indicates fear memory retrieval (early extinction CS+: RE+CORT: 0.13 ± 0.03; RE: 0.14 ± 0.03; CORT: 0.15 ± 0.04; early extinction CS−: RE+CORT: 0.12 ± 0.03; RE: 0.11 ± 0.02; CORT: 0.12 ± 0.02); the significant reduction of response to the CS+ (**p < 0.01) and the lack of difference between the stimuli at the later phase indicate successful fear extinction (late extinction CS+: RE+CORT: 0.08 ± 0.02; RE: 0.13 ± 0.03; CORT: 0.09 ± 0.02; late extinction CS−: RE+CORT: 0.09 ± 0.03; RE: 0.14 ± 0.03; CORT: 0.12 ± 0.03). As no interaction or main effect of group were found, the graph presents the groups combined (*N* = 74; RE+CORT group: *n* = 24, RE group: *n* = 25, CORT group: *n* = 25).

SCR data from the third testing day revealed that both initial fear retrieval, as well as fear extinction learning, were accomplished; no differences were found between the groups. We used repeated-measures ANOVA to compare the response to the CS+ and CS− at the early phase of extinction (mean of trials 1–5) to its late phase (mean of trials 6–10); Group was used as the between-subjects factor. An interaction of CS and Time (*F*_(1,71)_ = 10.21, *p* = 0.002) was found, following which we analyzed each CS separately for the effects of Time (with Group as between-subjects factor). We observed an effect of Time for the CS+ (*F*_(1,73)_ = 10.71, *p* = 0.002) with a reduction in SCR at the late phase of extinction compared with the early phase. For CS−, no effect of Time was found (*F*_(1,73)_ = 0.62, *p* > 0.1). In addition, we tested for CS effects in either the early phase or late phase of extinction (with Group as between-subjects factor). A significant CS effect was found in the early phase (*F*_(1,73)_ = 6.42, *p* = 0.013): with higher SCR to CS+ compared to CS−, this indicated fear memory retrieval. The lack of a CS effect at the late phase of extinction (*F*_(1,73)_ = 2.82, *p* = 0.1) indicated successful extinction learning. Figure 3 demonstrates fear retrieval at the beginning of the extinction training and the subsequent fear reduction at the end of extinction learning. Due to the lack of group differences (in either the early/late phases of extinction and in either the first/last extinction trials for both CS; all *p* > 0.1) and the lack of any group interactions (all *p* > 0.05), all groups are combined.

#### Reinstatement Test

Reinstatement occurs after an unsignaled presentation of the UCS (Haaker et al., 2014; Lonsdorf et al., 2017). In our design, after extinction training was completed, participants received four unsignaled shocks. Then, they were presented with the original CS (CS+, CS−) and their modified version (CS+M, CS−M). Due to this design, we performed separate analyses for the reinstatement test of the original stimuli and the modified stimuli.

**Figure 4:**
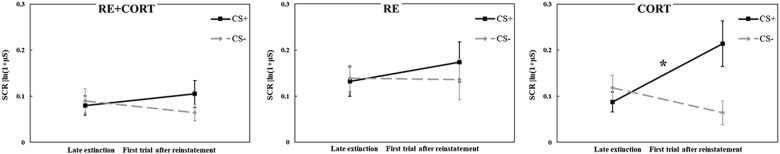
Day 3: Analyses of the reinstatement test to the original stimuli compared the mean (± SEM) SCR to the conditioned stimuli (CS+, CS−) in the late phase of extinction (mean of the last five trials; late extinction CS+: RE+CORT: 0.08 ± 0.02; RE: 0.13 ± 0.03; CORT: 0.09 ± 0.02; late extinction CS−: RE+CORT: 0.09 ± 0.03; RE: 0.14 ± 0.03; CORT: 0.12 ± 0.03) and the first trial after reinstatement (first reinstatement trial CS+: RE+CORT: 0.10 ± 0.03; RE: 0.17 ± 0.04; CORT: 0.21 ± 0.05; first reinstatement trial CS−: RE+CORT: 0.06 ± 0.02; RE: 0.14 ± 0.04; CORT: 0.06 ± 0.03) in the three experimental groups (*N* = 73; RE+CORT group: *n* = 24, RE group: *n* = 25, CORT group: *n* = 24). In the CORT group, differential reinstatement (i.e., higher SCR after the reinstatement shocks only to CS+) was found (*p < 0.05). No reinstatement was found in the two reactivation groups, RE+CORT and RE (all *p* < 0.01).

To examine the reinstatement effect of the original CS, a repeated-measures ANOVA with the within-subjects factor CS (CS+, CS−) and Time (mean of the last five trials of extinction, first trial after reinstatement shocks) and the between-subjects factor Group was performed. The analysis revealed a significant CS × Time × Group interaction (*F*_(2,70)_ = 3.62, *p* = 0.032). To investigate the origin of this interaction, we re-ran the ANOVA with the factors CS and Time in each group separately. No main effects of CS, Time or interaction of CS × Time were found in the RE+CORT and RE groups (all *p* > 0.1). However, the analysis revealed a significant CS × Time interaction (*F*_(1,23)_ = 13.42, *p* = 0.001) in the CORT group. To further investigate this interaction, ANOVA with the factor Time was then performed separately for CS+ and CS−. For CS+, a significantly higher response at the first trial of reinstatement compared to the late phase of extinction (*F*_(1,23)_ = 6.78, *p* = 0.016) demonstrated a reinstatement effect. For CS−, the analysis revealed a significant effect of Time (*F*_(1,23)_ = 6.26, *p* = 0.020). Here, the response was significantly lower at the first trial of reinstatement compared with the late phase of extinction. Figure 4 presents the reinstatement test for the original stimuli in the three groups, depicting the differential reinstatement effect in the no-reactivation (CORT) group and the lack of reinstatement in the two reactivation groups.

**Figure 5:**
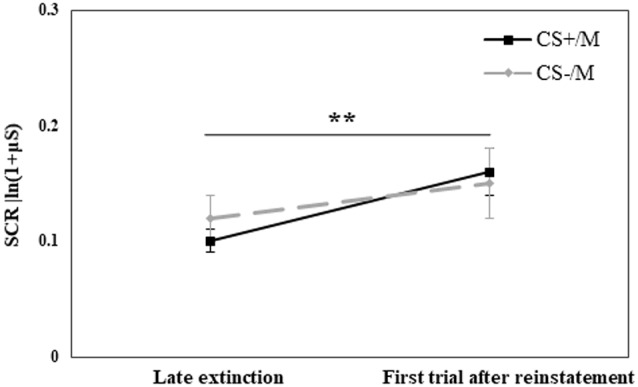
Day 3: Analyses of the reinstatement test to the modified stimuli compared the mean (± *SEM)* SCR to the stimuli in the late phase of extinction (mean of last 5 trials of the original CS+ or CS−; late extinction original CS+: RE+CORT: 0.08 ± 0.02; RE: 0.13 ± 0.03; CORT: 0.09 ± 0.02; late extinction original CS−: RE+CORT: 0.09 ± 0.03; RE: 0.14 ± 0.03; CORT: 0.12 ± 0.03) and the first trial after reinstatement (modified stimulus, CS+M or CS−M; first reinstatement trial CS+M: RE+CORT: 0.11 ± 0.03; RE: 0.18 ± 0.04; CORT: 0.18 ± 0.04; first reinstatement trial CS−M: RE+CORT: 0.16 ± 0.05; RE: 0.12 ± 0.04; CORT: 0.18 ± 0.05). The analysis revealed a generalized reinstatement (i.e., to both CS+M and CS−M; **p = 0.005). Since no group effect or interaction were found, the figure presents all three experimental groups combined (*N* = 73; RE+CORT group: *n* = 24, RE group: *n* = 25, CORT group: *n* = 24).

We then performed a similar analysis with the modified CS. We used a repeated-measures ANOVA with the within-subjects factor CS (CS+/M, CS−/M) and Time (using the original CS for the mean of the last five trials of extinction and the modified CS for the first trial after reinstatement shocks) and the between-subjects factor Group. The analysis revealed a main effect of Time (*F*_(1,70)_ = 8.31, *p* = 0.005), with a significantly higher response following the reinstatement shocks (for all additional comparisons, including group, *p* > 0.05). Figure 5 presents the reinstatement effect for the modified stimuli, due to the lack of group differences (all *p* > 0.05), all groups are combined.

To further explore differences in response patterns between original and modified stimuli, we analyzed the response to all four stimuli in the first trial of reinstatement. An ANOVA with the within-subjects factors Type (CS+ vs. CS−) and Size (original vs. modified) and the between-subjects factor Group revealed a significant interaction of Type and Size (*F*_(1, 70)_ = 4.18, *p* = 0.045). An analysis of the effect of Size (original vs. modified), performed separately on CS+ and CS−, revealed that size affected only the CS− (*F*_(1,70)_ = 7.58, *p* = 0.007) as the CS−M led to higher SCR than the original CS− at the first trial after reinstatement. An analysis of the effect of Type performed separately on the original and modified stimuli revealed that Type affected only the original stimuli (*F*_(1,70)_ = 13.10, *p* = 0.001) as only the original CS+ led to higher SCR compared to CS− at the first trial after reinstatement (not shown; for all additional comparisons, including group, *p* > 0.05). These results show that a modification of the CS− (but not the CS+) leads to an increase in SCR to this stimulus.

## Discussion

In this study, we aimed to elaborate on our previous findings on the cortisol-dependent enhancement of fear memory reconsolidation in men (Meir Drexler et al., 2015) using an alternative reactivation method (i.e., a low-intensity UCS presentation instead of unreinforced CS reactivation). We hypothesized that the combination of UCS reactivation and cortisol would lead to an enhanced and generalized fear memory reconsolidation, which would be manifested in higher SCR after reinstatement to both the original CS+ and stimuli that resemble it.

### Performance in the 3-Day Fear Conditioning Paradigm

The results confirmed successful fear learning on the first day without any baseline differences between the groups. In addition, no differences between the cortisol and placebo reactivation groups were seen in SCR to the reactivation shock on the second day, demonstrating that cortisol did not affect SCR during reactivation itself. On the third day, the results demonstrated fear retrieval in the first half of extinction followed by extinction learning towards the second half. Several reconsolidation studies demonstrated group differences between the control group/s and the reconsolidation group already during extinction training (Kindt et al., 2009; Schiller et al., 2010; Agren et al., 2012). However, in line with our previous reconsolidation-cortisol/stress studies (Meir Drexler et al., 2015, 2016; Meir Drexler and Wolf, 2017b), no group differences were seen at this phase in the current study as well.

Immediately after extinction and the presentation of four reinstatement shocks, SCR to the original stimuli and their modified versions were tested. While a reinstatement effect to the original stimulus CS1+ was found, as expected, in the no-reactivation (cortisol) group, no reinstatement effect was found in the two reactivation groups, regardless of the pharmacological treatment. The response to the modified stimuli, however, was different, with significantly higher SCR to both modified CS without any group differences.

### Low-Intensity UCS Reactivation Inhibits the Return of Fear

Our previous publication on the effects of cortisol on memory reconsolidation (Meir Drexler et al., 2015) revealed that cortisol has an enhancing effect on fear memory reconsolidation in men. Similar to other reconsolidation studies (e.g., Schiller et al., 2010), the memory modification we achieved in that study was limited to the reactivated CS+ and did not generalize to other stimuli that were not reactivated. As fear memories are often generalized (Dunsmoor and Murphy, 2015; Pollack et al., 2018), in the current study we aimed to achieve a more generalized effect of cortisol on fear memory reconsolidation using an alternative reactivation method, i.e., UCS presentation. Direct comparisons between CS and UCS reactivation have previously shown that UCS reactivation leads to a more generalized reconsolidation effect than CS reactivation (Liu et al., 2014; Luo et al., 2015). In a sharp contrast to our hypothesis, however, we found that UCS reactivation inhibited the return of fear, independent of cortisol.

Stress and GC (e.g., cortisol) are strong modulators of learning and memory processes (Wolf et al., 2016; de Quervain et al., 2017), including memory reconsolidation (Meir Drexler and Wolf, 2017c), but previous findings have reported some mixed findings on the direction of their effects (Akirav and Maroun, 2013; Meir Drexler and Wolf, 2017c). These conflicting findings may be a result of methodological differences. Memory related factors (type, strength, age of memory), individual differences, and manipulation-related factors can all influence whether or not a reconsolidation effect is achieved (the so-called “boundary conditions”) as well as its direction (Meir Drexler and Wolf, 2017c). The current study was similar in key methodological factors to that of our previous publication, in which cortisol-dependent reconsolidation enhancement was detected (Meir Drexler et al., 2015): type, strength, and age of memory (i.e., 1-day old fear memory), sampled population (i.e., men only), and overall design (e.g., 3-day paradigm, cortisol). The only methodological difference between the two studies was in the reactivation method, which is another key factor that can affect the strength and direction of a reconsolidation effect (Meir Drexler and Wolf, 2017c; Liu et al., 2019). Unlike the commonly used CS reactivation, the low-intensity UCS reactivation literature is rather limited. Nonetheless, prior works on UCS reactivation and on CS triggered post-retrieval counterconditioning suggest possible reasons for the findings we reported here.

#### UCS Reactivation Generates a Strong Prediction Error

UCS reactivation, in combination with additional treatment (mainly post-retrieval extinction), was found to inhibit the return of fear. For instance, Liu et al. (2014) compared CS reactivation and UCS reactivation, both followed by extinction training. Unlike reactivation by CS, UCS reactivation led to a more generalized effect. Namely, unextinguished CS+ (i.e., stimuli that were previously paired with the UCS but were never extinguished) were also impaired by UCS triggered extinction. This was not the case for CS triggered extinction. Moreover, both recent and remote memories were affected. These findings led the authors to conclude that reactivation by the UCS destabilizes all CS associations with that UCS, while CS reactivation specifically disrupts the association between the reactivated CS+ and the UCS. Findings in rats demonstrate a significant difference at the neural level as well, with UCS reactivation leading to stronger alterations (e.g., endocytosis of glutamate receptors, activation of protein kinase A in the hippocampus) compared with CS reactivation. Similar findings were shown by Luo et al. (2015) in their animal study on drug-seeking behavior: the original UCS was cocaine and the UCS used for reactivation was methylphenidate, a drug that mimics the effects of cocaine and thus leads to priming. In this instrumental task, UCS reactivation before extinction led to decreased renewal and reinstatement of previously reinforced behaviors, even if they were not directly extinguished. This effect was modulated by the regulation of endocytosis of AMPA receptors in the basolateral amygdala. When endocytosis of AMPA receptors was inhibited, the effect of UCS triggered extinction was prevented.

Interestingly, UCS reactivation, which leads to a non-specific effect on all related CS, seems to be specific to the UCS itself. When associations were created to more than one UCS (e.g., a painful stimulus to the eye or to the foot), only the UCS that was reactivated (and its corresponding CS) were affected by the manipulation (for instance, MAPK inhibition: Díaz-Mataix et al., 2011). This led the authors to suggest that once the association between UCS and its multiple predictors is created, the neural representation of these stimuli becomes a unique entity in the amygdala. Presenting either element of the association later may open the possibility for impairment of this association.

The robustness of the UCS reactivation procedure might thus stem from the larger prediction error it generates compared to CS reactivation. In CS reactivation, only a single association (CS1+/UCS) is violated. In contrast, since the UCS is initially paired with multiple CS (e.g., CS1+, CS2+, CS3+), all absent during the reactivation procedure, multiple associations are violated. The larger prediction error in UCS reactivation paradigms can affect memories that are usually more resistant to interferences, such as older and stronger memories (Liu et al., 2014, 2019; Dunbar and Taylor, 2017). For instance, Thompson and Lipp (2017) found that UCS reactivation and extinction leads to lower fear responses to both fear-relevant (spiders, snakes) and fear-irrelevant (geometric shapes) stimuli. This strong prediction error may thus explain why UCS reactivation affected both reactivation groups, regardless of cortisol. The direction of the effect, however, requires additional explanation.

#### Low-Intensity UCS Reactivation Alters UCS Valence

In contrast to the studies described above, our study included no behavioral manipulation (e.g., extinction) immediately following the UCS reactivation, and the pharmacological manipulation that was employed led to no group differences by itself, compared to placebo. The strong UCS-generated prediction error itself cannot account for an impairment in memory reconsolidation, as memory destabilization without interference (e.g., by anamnestic agent) is expected to lead to either memory preservation or strengthening, but not to an impairment (Exton-McGuinness et al., 2015; Exton-McGuinness and Milton, 2018; Sinclair and Barense, 2019).

The integration concept, suggested by Gisquet-Verrier and Riccio (2019), however, may account for our findings. According to this concept, reactivated memories are labile and can integrate new information. Depending on the information content, integration could either result in updating (e.g., the addition of new information), strengthening (e.g., promnesic treatments), disrupting (e.g., amnesic treatment or interference), or distorting (e.g., false information, counterconditioning) of the initial memory. In line with this concept, Liu et al. (2019) suggested that the UCS reactivation procedure leads to an update rather than to a disruption of the original memories. We thus suggest that the less negative consequences of the low-intensity UCS reactivation procedure were incorporated into the original fear memory, resulting in lower conditioned responses to the CS. The UCS reactivation literature currently does not present a similarly designed study that would allow a direct comparison supporting our findings. Mainly, the literature does not include a reactivation-only group. To support our conclusion, lower fear recovery should be seen in a UCS reactivation-only group using additional recovery indices (e.g., renewal, spontaneous recovery). In addition, more evidence is needed to support this effect in women, as we included only men in our study.

Nonetheless, additional findings may support our observation on the possible strength of the low-intensity UCS reactivation method. In a study by Popik et al. (2019), animals were trained to associate a tone with a strong footshock. Then, the original shock level was replaced with a weaker one, which was presented together with the CS in a procedure termed “deconditioning.” This led to a more long-lasting reduction of fear, resisting both renewal and spontaneous recovery, compared with traditional extinction training that lacks a presentation of the UCS altogether. These findings are in line with CS reactivation studies, in which reversal learning or counterconditioning were used as post-retrieval manipulation. In rats, for instance, Olshavsky et al. (2013) showed that post-retrieval fear learning can lead to the updating of an initial appetitive memory, promoting a reduction in appetitive behavior to the CS. In mice, Redondo et al. (2014) demonstrated a reversal of either fear memory or reward memory using conditioning of the opposite valence (i.e., either reward or fear, respectively) after optogenetic reactivation of the original memory engram in the hippocampus. In humans, Gera et al. (2019) found a significant reduction in the reinstatement of appetitive memory in a paradigm in which CS reactivation was followed by aversive counterconditioning (i.e., the CS was paired with a monetary loss instead of gain). In contrast to traditional counterconditioning methods (e.g., aversion therapy for drug addiction) in which a new memory trace is formed, this post-retrieval counterconditioning is thought to update the valence, valuation or salience of the original memory (Das et al., 2015; Goltseker et al., 2017). Due to the integration of new information in the original maladaptive memory, this method may be more successful in preventing relapse and thus have significant therapeutic implications (Gisquet-Verrier and Le Dorze, 2019).

### Therapeutic Implications and Limitations

In real-life situations, maladaptive memories are often associated with multiple cues. For a reconsolidation manipulation to be effective in the clinical sense, the reconsolidation disruption should be generalized to other associated stimuli, for instance, from one type of spider presented during treatment to another (Soeter and Kindt, 2015). In our previous studies, both the generalizability of the effect and its direction varied according to the manipulation (stress or cortisol: Meir Drexler et al., 2015; Meir Drexler and Wolf, 2017b). The reasons that led to a generalized reconsolidation effect in a CS reactivation design in some cases (Soeter and Kindt, 2015; Meir Drexler and Wolf, 2017b), but to a specific effect in others (Schiller et al., 2010; Hupbach and Dorskind, 2014; Meir Drexler et al., 2015) remains to be determined. In contrast, various UCS reactivation studies pointed to a generalized effect (Liu et al., 2014; Luo et al., 2015) and an improved ability to affect stronger, potentially more robust memories (Liu et al., 2014; Luo et al., 2015; Dunbar and Taylor, 2017). However, in the current study, using a UCS reactivation design, only the original stimulus was affected. This specific effect was possibly a result of the study design. Potentially, since the modified stimuli were not part of the original CS+/UCS association and since memory generalization might require time (Pollack et al., 2018), they were not affected by the reactivation of the UCS. The higher SCR to the modified stimuli can thus represent a merely enhanced response to potentially novel stimuli.

Given the limited literature on UCS reactivation, more research is needed to determine the necessary conditions for this paradigm to be successful in destabilizing multiple memory traces. For treatment, a direct reactivation of the original UCS might be challenging, and even more so, a quantitate reduction of its intensity. However, in cases that involve the intake of substances, direct exposure to the UCS and control over its amount or intensity might be easy to accomplish relative to fear-related learning. In rats, administration of the β-adrenoreceptor antagonist propranolol after UCS reactivation by a nicotine priming dose inhibited nicotine conditioned place preference and relapse after short or prolonged abstinence. Among people who smoke, propranolol led to a decrease in nicotine preference that was induced by both newly learned and existing (i.e., real-life) stimuli (Xue et al., 2017). Fortunately, some studies suggest that lower-intensity UCS reactivation is not mandatory for achieving a generalized effect. As prediction error does not necessarily require the absence of either stimulus, it can be also achieved by other violations of the learning history. For instance, Díaz-Mataix et al. (2013) found that a temporal error (i.e., alteration from the original CS+/UCS interval during fear acquisition training) can trigger memory reconsolidation as well. This method can then be used to disrupt multiple fear memory associations (e.g., using the protein synthesis inhibitor anisomycin). Whether a variation of this manipulation can lead to reconsolidation impairment by itself, as we demonstrated here with lower-intensity UCS reactivation, has to be determined in future studies.

## Conclusion

In this study, we aimed to extend our previous findings on the enhancing effects of cortisol on the reconsolidation of fear memories in men. For this aim, we used an alternative reactivation method, which involved a low-intensity UCS presentation (instead of the more commonly-used unreinforced CS reactivation) and usually leads to a more generalized effect. In contrast to our hypothesis, UCS reactivation prevented the return of fear regardless of the pharmacological manipulation. We conclude that the robust prediction error in combination with the lower UCS intensity has by itself weakened the emotional value of the UCS, thus preventing the return of fear to the CS associated with it. Further research is needed to support our findings and to adapt this reactivation method, or variations thereof, as a tool for therapeutic use.

## Data Availability Statement

The raw data supporting the conclusions of this manuscript will be made available by the authors, without undue reservation, to any qualified researcher.

## Ethics Statement

The studies involving human participants were reviewed and approved by the ethics committee of the Faculty of Medicine at the Ruhr University Bochum (registration number: 16-5788, approval date: 11/8/2016) and was conducted in accordance with the Declaration of Helsinki. The participants provided their written informed consent to participate in this study.

## Author Contributions

All authors (SM, CM, SL, MT, and OW) contributed to the design and implementation of the research, to the analysis of the results and to the writing of the manuscript.

## Conflict of Interest

The authors declare that the research was conducted in the absence of any commercial or financial relationships that could be construed as a potential conflict of interest.
